# *Salmonella *Typhimurium infection: Type I Interferons integrate cellular networks to disintegrate macrophages

**DOI:** 10.15698/cst2018.02.125

**Published:** 2018-01-30

**Authors:** Nirmal Robinson

**Affiliations:** 1 Cluster of Excellence in Cellular Stress Responses in Aging-associated Diseases (CECAD), University of Cologne, Cologne, Germany.; 2 Institute for Medical Microbiology, Immunology and Hygiene, University Hospital Cologne, Cologne, Germany.

**Keywords:** Salmonella, interferon, Nrf2, RIP, p62, PGAM5, macrophage, autophagy, cell death, necroptosis

## Abstract

Type I interferons have immunomodulatory functions during infection with bacteria and viruses. They are vital for the host defense against viruses and extracellular bacteria. However, recent evidences show that IFN-I contributes to immunopathology during intracellular bacterial infection. We had previously shown that IFN-I receptor knock out mice (*ifnar^-/-^*) are less susceptible to *S*. Typhimurium infection and the macrophages are resistant to *S*. Typhimurium-induced cell death dependent on RIP kinases commonly known as necroptosis. We have now recently shown that IFN-I-signaling through the activation of RIP kinases and PGAM5 exacerbates necroptosis in *Salmonella *Typhimurium-infected macrophages by downregulating Nrf2-dependent cytoprotective response mechanisms [Hos *et al*, JCB 2017].

* Salmonella* *enterica* serovar Typhimurium (*S.* Typhimurium) are facultative intracellular bacteria that effectively evade innate immune defense mechanisms using a plethora of pathogenic principles encoded in their pathogenicity islands namely *Salmonella* Pathogenicity islands. Systemic infection of the pathogen involves interactions with macrophages, which eliminate pathogens by various mechanisms including phagocytosis, autophagy, production of toxic free radicals and inflammasome activation. Nevertheless, *S.* Typhimurium redirects the cellautonomous immune responses to its own advantage. They have evolved strategies to survive in the hostile phagosomal-milieu of macrophages and replicate. The success of the pathogen has been attributed to its ability to induce profound inflammation. For instance, TLR sensing and stimulation are required to control the extracellular growth of *S. *Typhimurium, on the contrary TLR-activation promotes intracellular survival and growth of the bacteria. We had previously identified type-I interferons (IFN-I) that are induced through TLR activation as key inflammatory mediators of *S.* Typhimurium-induced pathogenicity in mice and the IFN-I receptor knock out (*ifnar*^-/-^) mice are less susceptible to infection. We had specifically demonstrated that IFN-I signaling in macrophages is a key mechanism by which *S.* Typhimurium establishes infection.

An important outcome of infections is the death of host cells. Cell death is beneficial to host in containing infection but *Salmonella* benefits from inducing cell death to disseminate. They engage cytosolic receptors NLRP3 and NLRC4 leading to caspase-1 activation and subsequent secretion of IL-1β and IL-18 which results in a form of cell death known as pyroptosis. *S.* Typhimurium can also induce an alternate form of cell death known as necroptosis regulated by receptor interacting protein kinase 1 (RIP1) and 3 (RIP3). We had shown that IFN-I signaling drives RIP1 activation in macrophages upon *S.* Typhimurium infection, which subsequently phosphorylates RIP3 and cell death follows. An interesting observation was that *ifnar^/-^* macrophages are resistant to cell death despite inflammasome activation. These findings raise important questions: Does pyroptosis and necroptosis occur in different subsets of macrophages or do they occur simultaneously upon IFNAR activation? Does IFN-I signaling integrate these cell death pathways during bacterial infection?

Macrophages subjected to IFN-I treatment do not undergo cell death, which indicates that during *S.* Typhimurium infection an additional trigger combined with IFN-I signaling culminates in necroptosis. *In vitro* experiments revealed that *ifnar^-/-^* macrophages are resistant to cell death despite the injury caused by *S.* Typhimurium is similar to that of the wild type (WT) macrophages. This indicates that *ifnar^-/-^* macrophages are able to recover from the injury caused by the pathogen and are capable of restoring homeostasis. This prompted us to investigate autophagy, a vital process in restoring cellular and energy homeostasis. Autophagy has also been associated with necroptosis although both its pro necroptotic and anti necroptotic roles have been reported in different cellular models and pathologies. Our recent findings revealed that* S.* Typhimurium-infected macrophages are defective in autophagy because, the critical regulators of autophagy namely AMPK and Sirt1 are degraded in the lysosomes. Moreover, autophagy-deficient *Atg7^-/-^* macrophages are more susceptible to *S.* Typhimurium-induced cell death. In this context, it will be interesting to determine if IFN-I-signaling promotes the lysosomal degradation of AMPK and Sirt1. Interestingly, we observed that autophagy was not induced in *S.* Typhimurium infected *ifnar^-/-^* macrophages. This implies that either autophagy is less required due to reduced mitochondrial damage or IFN-I-signaling positively regulates autophagy.

WT macrophages infected with *S.* Typhimurium also display enhanced expression of p62 but it is further pronounced in *ifnar^-/-^* macrophages consistent with the inhibition of autophagy. Evidences suggest that upon oxidative stress, p62 interacts with the Nrf2-binding site of Keap1 thus disrupting Nrf2-Keap1 interaction. Nrf2 is subsequently stabilized and translocated to the nucleus, where it binds to conserved antioxidant response elements (AREs) in the promoter regions of target genes. Consistent with increased generation of mitochondrial reactive oxygen species (ROS), Nrf2-dependent anti-oxidative responses are significantly reduced in *S.* Typhimurium-infected macrophages and it is dependent on IFN-I signaling.

Enhanced p62 expression provided us the initiative to investigate Nrf2-responsive anti-oxidative mechanisms. Nevertheless, it did not coherently explain the role of IFN-I signaling in reduced anti-oxidative response and necroptosis because p62 is also highly expressed in WT macrophages. Our previous findings have shown that IFN-I activates RIP kinases. Therefore, we surmised that the blunted Nrf2 response could be dependent on RIP kinase signaling. Interestingly, we observed that Nrf2 expression and its transcriptional function were enhanced in RIP3-deficient macrophages similar to that of *ifnar*^-/-^ macrophages.

The molecular factors downstream of RIP kinases in mediating necroptosis remain less characterized. Upon TNF-induced necroptosis, PGAM5 is recruited to RIP-1 and RIP-3 complex on the outer mitochondrial membrane, where it triggers Drp1-mediated mitochondrial fragmentation. Originally, PGAM5 was shown to bind Keap1 and it has also been shown to sequester Nrf2 in the cytoplasm. We now show that upon *S.* Typhimurium infection, IFN-I-signaling activates PGAM5 resulting in the sequestration of Nrf2 in the cytoplasm and subsequent degradation mediated by Keap1. This results in reduced anti-oxidative response, increased mitochondrial damage and cell death.

IFN-I-signaling in macrophages intersects with other mechanisms of cell death. Intriguingly, *S.* Typhimurium infected *ifnar^-/-^* macrophages produce IL-1β in enormity, which has been correlated with pyroptosis. It is currently unknown if IL-1β can be produced by independent mechanisms upon the loss of IFN-I-signaling. Our recent findings are consistent with previous reports that Nrf2 regulates IL-1β expression. Mitochondrial ROS-dependent cell death facilitated by IFN-I-signaling coincides with another form of cell death known as ferroptosis, which is caused by the accumulation of lipid based ROS. It remains to be investigated if IFN-I-regulated RIP-signaling also interferes with glutathione peroxidase that repairs oxidized lipid species. Thus, IFN-I-signaling in *S.* Typhimurium-infected macrophages integrates various cell-autonomous immune mechanisms such as inflammatory pathways modulated by JAK/STAT-signaling, autophagy, RIP-signaling and anti-oxidative responses, which eventually culminate in the disintegration of macrophages (**Figure 1**). However, the outstanding question is how these cross-regulatory networks are integrated by IFN-I signaling.

**Figure 1:**
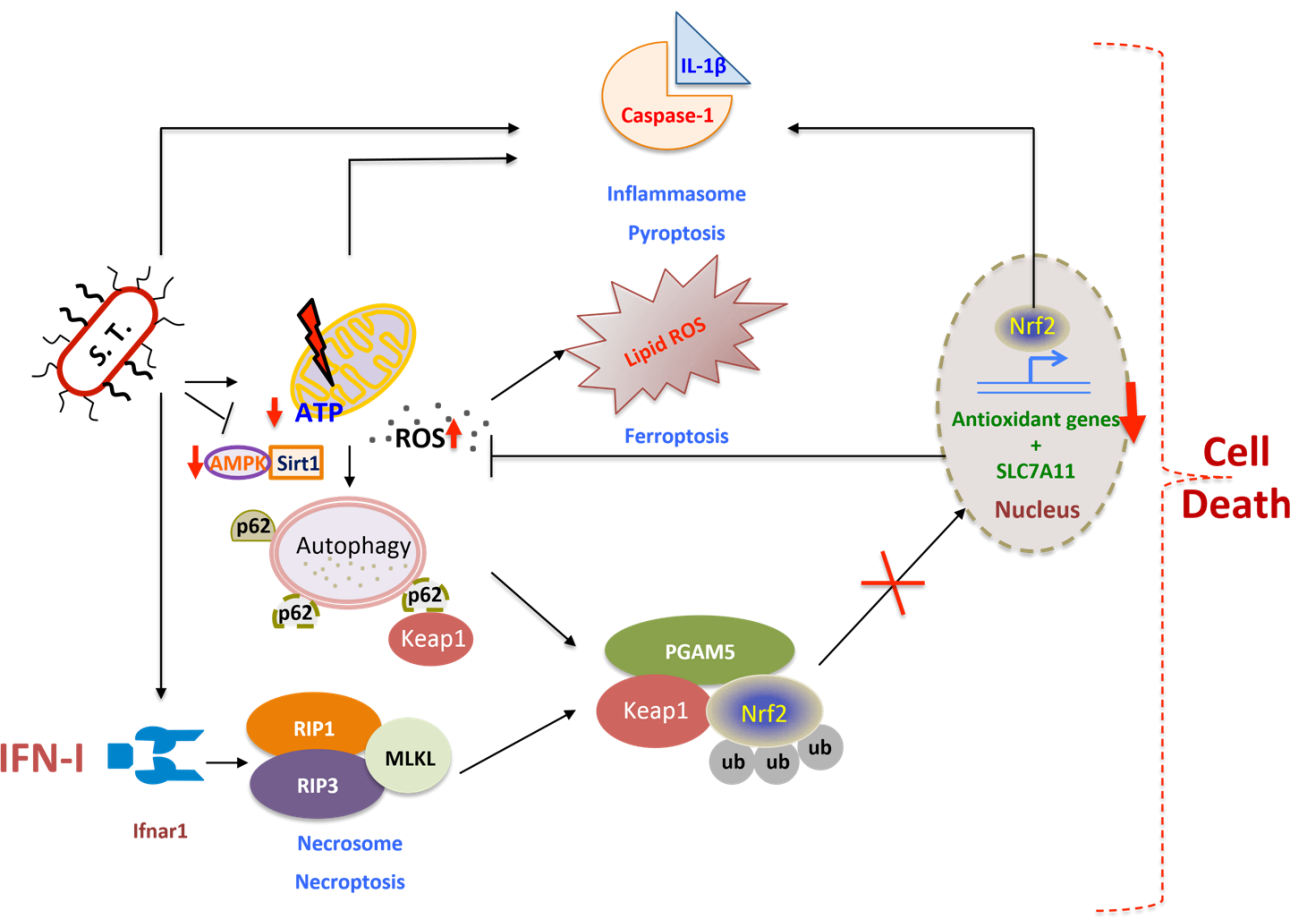
Figure 1: IFN-I integrates cellular networks to induce macrophage-death upon *S.* Typhimurium infection. *S. *Typhimurium (S.T) infection in macrophages results in mitochondrial damage hence reduction in ATP and mitochondrial ROS accumulation. This is a trigger for autophagy, however S.T abrogates AMPK and Sirt1 resulting in the inhibition of autophagy and p62 accumulation, which could sequester Keap1 enabling nuclear translocation of Nrf2 and transcription of cytoprotective anti-oxidative genes. However, type I interferons (IFN-I) induced by S.T. activates RIP kinases-mediated necrosome that subsequently activates PGAM5. PGAM5 sequesters Nrf2 in the cytoplasm and enables proteosomal degradation mediated by Keap1. Nrf2 is also known to regulate IL-1β expression, which is correlated with inflammasome activation and pyroptosis. Another cell death pathway termed as ferroptosis is also dependent on lipid based ROS regulated by SLC7A11 which is under the transcriptional control of Nrf2. Thus IFN-I integrates various cross-regulatory cellular networks, which eventually leads to the death of *S.* Typhimurium-infected macrophages.

